# Post-acute Sequelae of SARS-CoV-2 Infection: A Neglected Public Health Issue

**DOI:** 10.3389/fpubh.2022.908757

**Published:** 2022-06-17

**Authors:** Zhonglei Wang, Liyan Yang

**Affiliations:** ^1^Key Laboratory of Green Natural Products and Pharmaceutical Intermediates in Colleges and Universities of Shandong Province, School of Chemistry and Chemical Engineering, Qufu Normal University, Qufu, China; ^2^School of Pharmaceutical Sciences, Tsinghua University, Beijing, China; ^3^School of Physics and Physical Engineering, Qufu Normal University, Qufu, China

**Keywords:** post-acute sequelae of SARS-CoV-2 infection, COVID-19, public health issue, cardiovascular complications, metabolic abnormalities, multi-disciplinary PASC collaboration

## Introduction

The COVID-19 pandemic has caused at least 508,827,830 infections and is associated with a 1.2% mortality rate worldwide (1). New SARS-CoV-2 variants have driven new waves of the pandemic as a result of their increased transmissibility and ability to evade the immune response (2). The post-acute sequelae of SARS-CoV-2 infection (PASC) is an important but underestimated public health issue that can have a long-term impact on pulmonary and multiple extrapulmonary tissues and organs through several potential mechanisms (3, 4). Recent studies demonstrate that approximately 4–69% of patients (including children, adolescents, adults, and senior) suffer from PASC (5–11). There is considerable evidence concerning post-acute sequelae that will likely outlast the current pandemic and need to be addressed. This article reviews the clinical sequelae of COVID-19 survivors and provides valuable insights required to fill the gaps in medical knowledge.

## Pulmonary and Extrapulmonary Organ Sequelae

**Figure 1:**
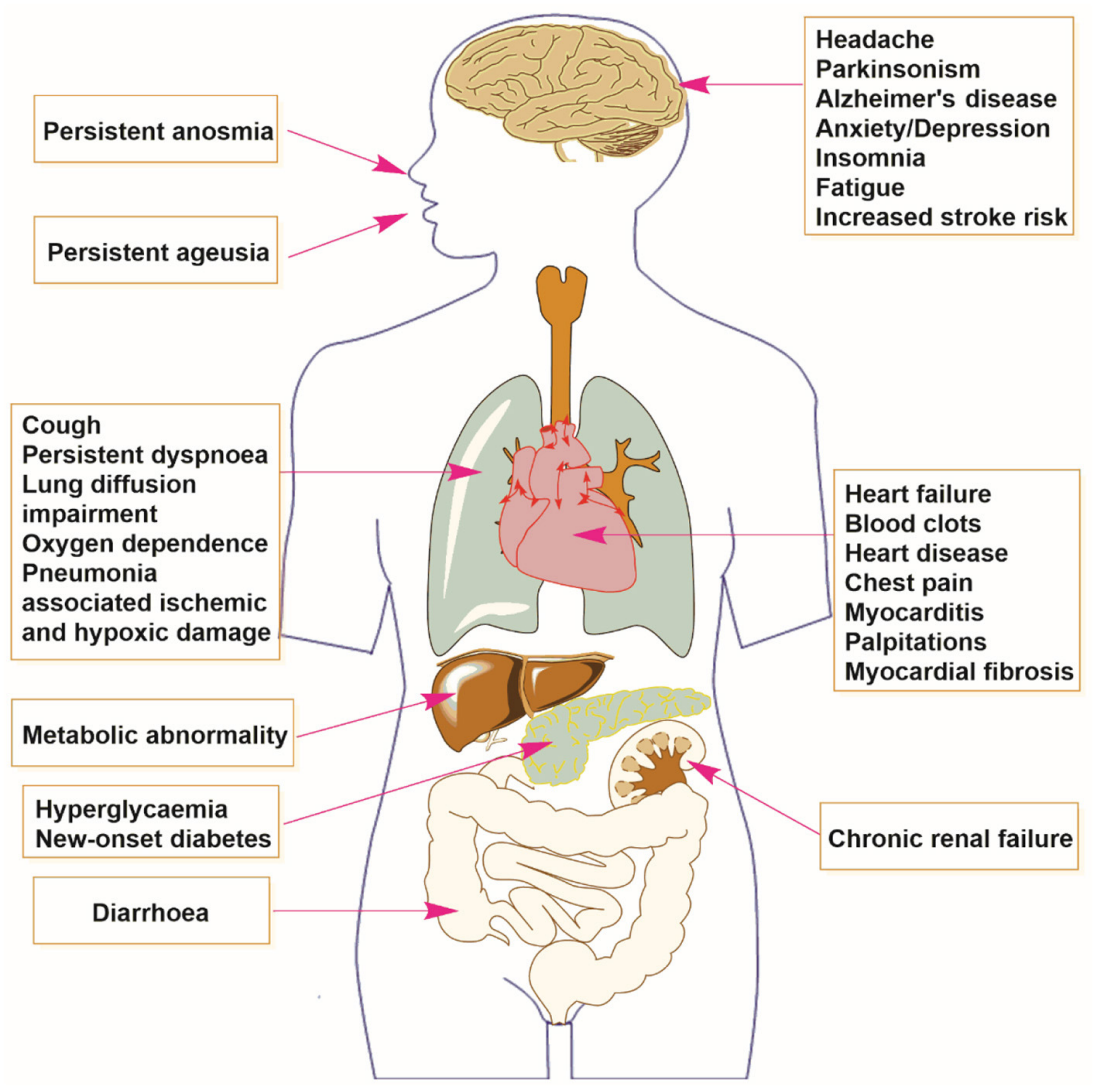
Multiple organ sequelae of SARS-CoV-2 infection.

There are several persistent sequelae occurring among COVID-19 survivors (see Figure 1). A longitudinal cohort study from Wuhan, China found that 1 year after COVID-19 diagnosis, 26% (313/1,185) and 30% (380/1,271) of survivors experienced dyspnea, or persistent breathlessness, at 6 and 12 months, respectively (12). The same study found that lung diffusion impairment was common among critically ill patients at 12 months (12). In a multicenter UK study, Evans et al. (13) found that of 1,077 hospitalized patients, 41% experienced dyspnea and 21–28% experienced palpitations and chest pain 5.9 months after discharge. A random-effect meta-analysis of 257,348 patients revealed that 25, 21, and 31% of survivors displayed persistent dyspnea at 6–8, 9–12, and >12 months follow-up, respectively (14).

There is an increased long-term risk of cardiovascular complications such as heart failure among patients with SARS-CoV-2 infection, even among mild cases (15). A large-scale study including a cohort of 153,760 COVID-19 survivors, an age-matched control group of 5,637,647 individuals, and a historical comparison group of 5,859,411 individuals, was conducted by Al-Aly et al. (16) to estimate the risk of cardiovascular sequelae. COVID-19 survivors had a significantly increased risk of cardiovascular disease within 1 year, including a 52% and a 72% increased risk of stroke and heart failure, respectively (16). SARS-CoV-2 infection is correlated with “new-onset” cardiovascular disease following infection (17). Rizvi et al. (18) independently reported that SARS-CoV-2-infected golden Syrian hamsters had cardiovascular complications such as ventricular wall thickening and interstitial fibrosis with elevated cardiac troponin I during the late phase of infection. Maio et al. (19) reported that the risk of thromboembolic events 8.5 months after the follow-up of COVID survivors (1.53%, *n* = 6,937) was five times higher than among population controls (0.31%, *n* = 435,104). An online survey showed that 53 and 68% of patients reported chest pain and palpitations 7 months after COVID-19 infection (20).

SARS-CoV-2 infection even among those with mild symptoms can cause severe cognitive and neurological defects (21). Recent studies have demonstrated that >10% of patients experience COVID-19-associated anosmia (21). A large UK-based community cohort study with 4,999 participants conducted from June 2021 to January 2022 found that patients infected with the omicron variant more frequently possessed a loss of smell than those infected with the delta variant (52.7 vs. 16.7%, respectively; *p* < 0.001) (22). Zazhytska et al. (23) found non-cell-autonomous disruption of olfactory sensory neuron nuclear architecture and down-regulation of olfactory receptors and signaling genes in SARS-CoV-2-infected hamster and human autopsies. These findings provide a potential pathophysiological mechanism linking COVID-19 and anosmia. Kraus et al. (24) provided an alternate mechanism by which the intranasal receptor-binding domain of SARS-CoV-2 spike protein causes olfactory receptor damage and olfactory system dysfunction in SARS-CoV-2-infected zebrafish. This finding has potential implications for the intranasal treatment of PASC. Douaud et al. (25) conducted a large-scale longitudinal neuroimaging cohort study of the brain images from 401 COVID-19 cases 51 to 81 years of age and 384 age-matched controls to estimate how changes to brain structure and function correlate with the taste and smell of infected patients. COVID-19 survivors showed a greater reduction in the gray matter thickness of the parahippocampal gyrus and entorhinal cortex, ranging from ~0.2 to ~2%, and a greater reduction in the global brain volume than controls (25).

To date, from anosmia, headaches, to Parkinsonism, Alzheimer's have been attributed to SARS-CoV-2 infection (26). A clinical study indicated that the risk of dementia was 2–3-fold higher among SARS-CoV-2-infected individuals than healthy controls (27). Semerdzhiev et al. (28) found that Parkinsonism is caused by a direct interaction between the SARS-CoV-2 N-protein and α-synuclein. Lang et al. (29) indicated that hypoxemia, or respiratory compromise, along with potential virus-specific endothelial mechanisms may account for post-infectious Parkinsonism. Revere et al. (30) found that Alzheimer's is associated with a higher expression of Angiotensin-Converting Enzyme 2 in the brains of COVID-19 survivors, and Shen et al. (31) showed that SARS-CoV-2 enters the brain, induces an Alzheimer's-like gene program in healthy neurons and exacerbates disease-related neuropathology. Fernández-de-las-Peñas et al. (32) found that 8.4–15% of COVID-19 survivors suffer from post-COVID headaches 6 months after infection.

“Long COVID” can cause metabolic abnormalities and immunological dysfunction (33–35). For example, in a cohort study of 551 discharged COVID-19 survivors in Italy, 35 and 2% had hyperglycemia and “new-onset diabetes,” respectively, after 6 months (33). In another retrospective England-based cohort study of 47,780 COVID-19 patients with a mean of 65 years of age, 2.9% had “new-onset diabetes” 4.6 months following infection (34). Thus, SARS-CoV-2 infection can cause multiple organ failure and induce long-lasting post-COVID sequelae that are of great concern.

## Discussion

The COVID-19 pandemic is ongoing and promising curative treatments do not yet exist (36, 37). Meanwhile, the sequelae of this infection have posed a considerable threat to global health and economic development. Considering the available evidence, additional preventive and treatment strategies are needed.

Current prophylactic measures, such as wearing masks and increasing vaccination coverage, are still necessary. Vaccination is associated with a lower risk of several COVID-19 sequelae and remains the most practical approach to preventing the further spread of the virus (38). After 2 years, 11,438,720,838 doses of the COVID-19 vaccine have been administered globally to combat SARS-CoV-2 infection (1). Third and even fourth vaccine booster doses are being administered in many countries to improve immunity (39). However, many low-income nations are still waiting to offer the initial doses (1). Vaccine inequity has enabled SARS-CoV-2 to spread rapidly, increasing the incidence of sequelae, and undermining global COVID-19 recovery efforts (40). Fair allocation of vaccines is critical for effective COVID-19 control and elimination in resource-limited settings. Fortunately, more countries are taking further action. In November 2021, President Xi announced that China would provide 1.0 billion, including 600 million donated, COVID-19 vaccine doses to African countries to help reach its goal of vaccinating 60% of its population by 2022 (41). Countries will need to collaborate to create a fairer vaccination environment required to bolster worldwide immunity.

In addition, therapeutic regimens, including small-molecule inhibitors and traditional medicine, are still needed. Small-molecule inhibitors are being widely studied and play an essential function in COVID-19 treatment. Gilead's controversial drug, Veklury^®^, was conditionally approved by the Food and Drug Administration (FDA) to combat the pandemic (42, 43) and Pfizer's oral broad-spectrum candidate, Paxlovid^®^, and Merck's oral prodrug, Lagevrio^®^, provide new hope for a COVID-19 cure (44). Even with promising clinical results, however, widespread use of these treatments may increase the virus' resistance to inhibitors. Researchers will need to carefully design more aggressive and effective strategies to address therapeutic limitations and uncertainties. For example, multi-target drug combination therapy (PF-07321332 + Remdesivir, Linoleic acid + Remdesivir, PF-07321332 + Molnupiravir), could enhance synergistic anti-COVID-19 efficacy while also reducing drug resistance (45). Traditional medicine is another valuable tool that should be considered for COVID-19 treatment. Many studies have shown that herbal medicine offers multi-organ protection against SARS-CoV-2 (46). Ye et al. (47) illustrated that licorice-saponin A3 and glycyrrhetinic acid, triterpenoids isolated from Gan-Cao, have strong inhibitory potency against SARS-CoV-2 infection at EC_50_ values of 75 nM against the SARS-CoV-2 nsp7 protein and 3.17 μM against the Spike protein. In the COVID-19 era, small-molecule inhibitors and traditional medicine have a distinct advantage and should be shared between laboratories.

PASC rehabilitation measures, such as multi-disciplinary PASC collaboration, are also critical. A database that includes the physiology, serological, clinical imaging, and epidemiological characteristics of PASC is required to better understand the condition. In addition, fundamental science research, including an understanding of the mechanisms of viral replication, disease pathogenesis, and host immunity is required to direct the earlier evaluation and future rehabilitation of survivors. Healthcare professionals will need to recognize and document pulmonary complications to improve mental and physical health by providing timely team-based, high-quality rehabilitation nursing to survivors. In short, clinical trials of the PASC and additional anti-PASC treatment options are required to fully understand and address this medical issue.

## Author Contributions

ZW: conceptualization, writing—original draft, writing—review and editing, visualization, and funding acquisition. LY: conceptualization, writing—review and editing, and funding acquisition. All authors contributed to the article and approved the submitted version.

## Funding

This work was supported by the project of the Ph.D. research start-up fund of Qufu Normal University, China (Grant Nos. 614901 and 615201).

## Conflict of Interest

The authors declare that the research was conducted in the absence of any commercial or financial relationships that could be construed as a potential conflict of interest.

## Publisher's Note

All claims expressed in this article are solely those of the authors and do not necessarily represent those of their affiliated organizations, or those of the publisher, the editors and the reviewers. Any product that may be evaluated in this article, or claim that may be made by its manufacturer, is not guaranteed or endorsed by the publisher.
